# Dysautonomia: a common comorbidity of systemic disease

**DOI:** 10.1007/s12026-025-09661-2

**Published:** 2025-07-08

**Authors:** Svetlana Blitshteyn

**Affiliations:** 1 Department of Neurology, Jacobs School of Medicine and Biomedical Sciences, University at Buffalo, 955 Main Street Buffalo, Buffalo, NY USA; 2 Dysautonomia Clinic, Williamsville, NY USA

**Keywords:** Postural orthostatic tachycardia syndrome (POTS), Neurocardiogenic syncope (NCS), Orthostatic hypotension (OH), Dysautonomia, Autonomic dysfunction

## Abstract

Referring to a broad spectrum of the autonomic symptoms, autonomic disorders, and general dysfunction of the autonomic nervous system, dysautonomia is one of the common and under-recognized comorbidities of a wide variety of systemic disease, including diabetes, autoimmune disorders, vitamin deficiencies, and hormonal dysregulation. The most common autonomic disorders encountered in clinical practice are postural orthostatic tachycardia syndrome (POTS), neurocardiogenic syncope (NCS), and orthostatic hypotension (OH), which may be undiagnosed or often mislabeled with psychiatric disorders. Typical clinical features of dysautonomia, such as orthostatic dizziness/lightheadedness, orthostatic intolerance, palpitations, exercise intolerance, cognitive dysfunction, and fatigue, should prompt a diagnostic investigation for dysautonomia, which includes an in-office 10-min stand test or a tilt table test in conjunction with other autonomic function tests if available. Treatment approach consists of non-pharmacologic and pharmacologic therapies with beta blockers, midodrine, ivabradine, pyridostigmine, fludrocortisone, stimulants, and other medications. In clinical setting, dysautonomia may present a diagnostic and therapeutic challenge in patients with various systemic disorders and may require a high index of suspicion on the part of the clinician. Importantly, diagnosing and treating dysautonomia is critical to providing comprehensive and personalized medical care to complex patients with chronic illness, who are typically highly symptomatic with multi-systemic complaints as a result of comorbid, and often undiagnosed, dysautonomia.

## Defining dysautonomia

Dysautonomia—defined here as autonomic dysfunction of any type—refers to abnormal function and disorders of the autonomic nervous system and encompasses a wide variety of autonomic symptoms, disturbances, and disorders. Many types of dysautonomia are common, such as neurocardiogenic syncope, POTS, orthostatic hypotension and orthostatic intolerance, but some are rare, such as familial dysautonomia and multiple system atrophy [1, 2]. Traditionally, dysautonomia has not been endorsed as a preferred term by some authorities because it does not signify a diagnosis or a disorder and is typically viewed as an umbrella category [3]. However, over the last few years, especially with SARS-CoV-2 pandemic, the term “dysautonomia” has been widely used to describe autonomic dysfunction as a common sequela of SARS-CoV2 infection and a major component in the pathophysiology of Long COVID and other neurologic disorders [4–6]. This overview will discuss dysautonomia as a common comorbidity of systemic disease in the context of clinical practice.

While objective diagnostic confirmation of the autonomic dysfunction is obviously preferrable, limited availability of and access to selected autonomic laboratories in the world have made such requirements for diagnosis problematic. It also necessitated the development of simplified, modified and user-friendly diagnostic criteria and classification system that can be easily applied in clinical setting by clinicians of all specialties. The diagnostic criteria have been established for common autonomic disorders, such as postural orthostatic tachycardia syndrome (POTS), neurocardiogenic syncope (NCS), orthostatic hypotension (OH) and inappropriate sinus tachycardia (IST) (Table 1) [1, 2]. However, when patients do not qualify for these diagnoses while exhibiting clinical features typical of these disorders, terms such as “autonomic dysfunction,” “orthostatic intolerance,” and “dysautonomia” are commonly used. Importantly, some neurologists believe that if the autonomic nervous system reacts appropriately to a stimuli, such as, for example, in a vascular compression syndrome or hypovolemia, that the term “autonomic dysfunction” and “dysautonomia” are incorrect because the autonomic nervous system is intact and reacts appropriately to an altered physiologic state. These assertions may be justified in the context of a detailed assessment of the autonomic nervous system via quantitative comprehensive autonomic function testing. Given the limited availability and accessibility to the autonomic laboratories, many physicians and patients are left without concrete diagnosis leading to missed diagnoses or misdiagnosis. Additionally, autonomic disorders may be missed when they do not qualify for common autonomic disorders based on a tilt table test or a 10-min stand test [7]. Similarly, patients with undiagnosed autonomic disorders may be mislabeled with various psychological or psychiatric disorders, resulting in wrong diagnostic and therapeutic approaches and missed opportunities to treat dysautonomia [8].

**Table 1:** Diagnostic criteria for common autonomic disorders

Autonomic disorder	Diagnostic criteria
POTS^1,2^	1. HR increase ≥ 30 bpm within 10 min for adults (≥ 40 bpm for adolescents 12–19 years of age) of standing or TTT
	2. Absence of OH
	3. Symptoms of orthostatic intolerance for ≥ 3 months
	4. Exclusion of other causes of postural tachycardia, such as dehydration, medication side effect and other medical conditions
NCS^1,2^	1. Transient loss of consciousness typically preceded by prodromal symptoms and signs, such as pallor, diaphoresis, nausea, abdominal discomfort, yawning, sighing, and hyperventilation. that may occur up to 60 s prior to loss of consciousness
	2. A sudden fall in blood pressure, heart rate and cerebral hypoperfusion on standing or TTT
OH^2^	Sustained drop in blood pressure ≥ 20/10 mmHG within 3 min of standing or TTT
IST^1^	1. Average sinus HR exceeding 90 bpm over 24 h or HR while awake and at rest ≥ 100 bpm
	2. Palpitations and other distressing symptoms associated with sinus tachycardia

While primary autonomic disorders have been given significant attention in neurology and neurological subspecialties, autonomic dysfunction in association with systemic disorders has not been emphasized. As a result, millions of patients with a wide variety of systemic disorders, ranging from post-acute infectious syndromes and autoimmune disorders to rare genetic disorders, and associated dysautonomia remain undiagnosed, misdiagnosed and untreated. To this end, dysautonomia represents a unique unmet need in patient care and is a major cause of widespread disability, functional impairment and substantial healthcare expenditure. Addressing these issues will require investment of resources in education and training of physicians of all specialties and developing clinical guidelines for practicing physicians on how to evaluate and manage patients with dysautonomia associated with systemic disease [6].

## Dysautonomia and systemic disease

Though not commonly acknowledged or recognized, autonomic dysfunction, referred here as dysautonomia, is a common manifestation of systemic disease and can be associated with a wide variety of disorders, hormonal changes, vitamin deficiencies, pharmaceutical and toxin exposure and injury (Table 2). Dysautonomia may be primary and may present in the form of POTS, NCS, OH or IST, or it can also manifest in undifferentiated autonomic dysfunction and orthostatic intolerance without meeting specific diagnostic criteria for primary autonomic disorders. Dysautonomia can also be secondary to or associated with various neurologic and medical disorders with different pathophysiologies, such as autoimmune and inflammatory, neoplastic, toxic, immunologic and post-acute infectious (Table 2). These associations have been demonstrated when patients are assessed via quantitative autonomic function tests and/or Composite Autonomic Symptom Score-31 (COMPASS-31), which is a validated questionnaire used to quantify the autonomic symptom burden [9]. Additionally, better diagnostic tests that assess and quantify cerebral perfusion highlight that autonomic dysfunction, or dysautonomia, with and without tachycardia may be a spectrum of the same disease [7].

**Table 2:** Classification of dysautonomia in clinical practice

Primary	Associated with neurologic disorders	Associated with systemic disorders	Associated with hormonal changes	Associated with vitamin deficiencies	Associated with pharmaceuticals	Associated with toxins	Associated with infection	Associated with injury or trauma
POTS	Migraine	EDS/HSD	Menarche	Vitamin B12 deficiency	Chemotherapy	Pesticides	COVID-19	Concussion
NCS	SFN	Autoimmune (Sjogren’s, celiac, undifferentiated, with known and unknown antibodies)	Pregnancy	Vitamin B1 deficiency	Anesthetics	Heavy metals	Influenza	Traumatic Brain Injury
NOH	CIDP/AIDP	MCAS	Perimenopause	Vitamin B6 deficiency	Some oral medications (stimulants, diuretics, SNRIs, quinolones)	Silicone	EBV and CMV	Spinal Cord Injury
IST	MS	Vascular compression syndromes	Pre-eclampsia	Iron deficiency	HPV vaccines	Cocaine	Lyme and co-infections	Whiplash
PAF	MG	Amyloidosis	Diabetes	Vitamin D3 deficiency	COVID vaccines	Alcohol	Enterovirus	Surgery
AAG	Autistic spectrum disorders	Inflammatory (Crohn’s)	Testosterone deficiency	Folate deficiency		Mold	Parvovirus	Invasive procedures
MSA	Stroke	Cancer (Paraneoplastic)	Adrenal insufficiency	Coenzyme Q10 deficiency		Air pollution	Streptococcus	Over-training for athletes
FD	Parkinson disease	Cardiovascular (HTN, CHF)	Thyroid disorders	Copper deficiency		Food additives, dyes, corn syrup	Pneumococcus	Lightening strike
Amyloid neuropathies	Polyneuropathy	Genetic (Fabry’s, hATTR and other unknown mutations)	Pituitary dysfunction			Energy drinks	Mycoplasma	Severe psychological stress
Hyperhidrosis and hypohidrosis	Sleep disorders	Mitochondrial disorders (known and unknown mutations)				Microplastics		PTSD

Abbreviations: *POTS* postural orthostatic tachycardia syndrome, *NCS* neurocardiogenic syncope, *NOH* neurogenic orthostatic hypotension, *IST* inappropriate sinus tachycardia, *PAF* pure autonomic failure, *AAG* autoimmune autonomic ganglionopathy, *FD* familial dysautonomia, *SFN* small fiber neuropathy, *CIDP*/*AIDP* chronic inflammatory demyelinating polyneuropathy/acute inflammatory demyelinating polyneuropathy, *MS* multiple sclerosis, *MG* myasthenia gravis, *EDS/HSD* Ehlers-Danlos syndrome/hypermobility spectrum disorders, *MCAS* mast cell activation syndrome, *HTN* hypertension, *CHF *congestive heart failure, *HPV* human papillomavirus, *EBV* Epstein-Barr virus, *CMV* cytomegalovirus, *PTSD* post-traumatic stress disorder

Clinical suspicion for dysautonomia should be raised in patients with autoimmune and inflammatory disorders, immunologic, hormonal and post-acute infectious syndromes who present with the following key features:

- Orthostatic dizziness/lightheadedness
- Orthostatic intolerance
- Exercise intolerance
- Palpitations
- Chronic fatigue
- Cognitive impairment (aka “brain fog”)

It is important to emphasize that although all 6 clinical features are commonly present, patients may not have all manifestations. Usually, patients with dysautonomia have had significant difficulty functioning despite treatment of their underlying medical condition and report physical and/or mental fatigue as their most disabling complaint. Importantly, most patients experience exercise intolerance or decreased exercise capacity compared to their pre-illness baseline state. Unfortunately, misdiagnosis with psychological and psychiatric disorders are common [8]. For example, in a large survey of patients with POTS, 80% reported misdiagnosis with anxiety or “all in your head” label [10]. Additionally, a large number of patients may carry a diagnosis of chronic fatigue syndrome and/or fibromyalgia: although autonomic dysfunction is a feature of both conditions, dysautonomia may not be diagnosed or treated in many of these patients, thus resulting in unmanaged debility and persistent disability for a condition with established diagnostic and therapeutic pathways [11, 12].

## Illustrative case

A 35-year-old woman with a 10-year history of celiac disease presented with dizziness, palpitations, difficulty concentrating and fatigue for 2 years. She has been on a strict gluten-free diet since her diagnosis of celiac disease and has not changed her diet or personal products to suspect contamination with gluten. Her gastrointestinal evaluation has not revealed worsening of celiac disease or a new gastrointestinal condition. A comprehensive metabolic panel, complete blood cell count and ferritin levels were all in the normal range. She was seen by a cardiologist who obtained ECG, 2D-cardiac echocardiogram and a 48-h Holter monitor, all of which were unremarkable. The episodes of palpitations were consistent with sinus tachycardia of 140 bpm on a Holter monitor with no evidence of cardiac arrhythmia. For difficulty concentrating (aka “brain fog”) and fatigue, she was evaluated by a neurologist and was found to have no evidence of demyelinating disease based on MRI of the brain, seizures based on EEG, or neuromuscular disorder based on an unremarkable neurologic exam. Her primary care physicians suggested that she may be depressed and anxious, and while she had no history of psychiatric illness, she agreed that her debilitating physical symptoms are making her depressed and anxious. She was started on sertraline 25 mg daily, but experienced significant nausea and dizziness, and as a result, discontinued it.

She subsequently sought out an autonomic specialist after checking her symptoms online and wondering if she may have POTS. The autonomic specialist performed an in-office 10-min stand test that showed a supine heart rate of 75 bpm that increased to 120 bpm after standing for 5 min with associated dizziness and palpitations. Blood pressure was 110/70 mmHG and remained unchanged, both in supine and standing positions. The patient was diagnosed with POTS based on clinical features and results of a 10-min stand test demonstrating a heart rate elevation by more than 30 bpm from supine to standing position (Table 1). She was encouraged to increase fluid intake to at least 2.5L per day and sodium chloride (table salt) intake to at least 7 g per day, which she had already implemented after reading about POTS online and prior to seeing an autonomic specialist. Atenolol 12.5 mg twice a day was started for postural tachycardia, which resulted in significant improvement in palpitations and fatigue. However, the patient continued to experience dizziness and difficulty sitting for prolonged periods of time at her job. At a follow-up visit, midodrine 2.5 mg three times daily was added, which was later increased to 5 mg three times daily for POTS. The patient reported significant improvement in dizziness and her orthostatic tolerance though she continued to experience fatigue and brain fog and required frequent breaks for hydration and rest.

The patient had dysautonomia in the form of POTS, which can be associated with celiac disease [13, 14]. Conversely, patients with celiac disease may have autonomic dysfunction and other neurologic manifestations [15]. Importantly, a gluten-free diet may not necessarily prevent the development of autonomic and other neurologic symptoms in a subset of patients commonly encountered in clinical practice.

Important points of care to consider in patients such as these are:

- Dysautonomia must be clinically suspected by physicians of all specialties involved in the care of patients with clinical features suspicious for autonomic dysfunction, including the internist, gastroenterologist, cardiologist, neurologist and others.
- Misdiagnosis with psychiatric disorders and treatment with antidepressants are common and need to be avoided unless there is clear evidence of major depression or generalized anxiety disorder that must be addressed.
- An in-office 10-min stand test is an important diagnostic test that can identify and confirm an autonomic disorder (Table 1), but if the stand test is negative, it does not exclude dysautonomia that may still be presented based on COMPASS-31 questionnaire or a tilt table test.
- Therapeutic modalities instituted for dysautonomia include non-pharmacologic measures, but implementation of pharmacotherapy is essential to autonomic symptom burden reduction. Pharmacotherapy should not be delayed and should be started at the initial visit if the patient reports that non-pharmacologic measures are ineffective or only partly effective.

## What is autonomic nervous system?

The autonomic nervous system (ANS) consists of sympathetic, parasympathetic and enteric divisions and is involved in numerous physiologic functions, including cardiovascular control of heart rate and blood pressure, gastric motility and secretion, bladder function, respiration, temperature control, and distribution of blood flow to organs and tissues. The ANS mediates the “flight or fight” response to both external and internal stimuli in order to maintain homeostasis [16]. There is evidence that the ANS is intimately involved in the process of inflammation as the vagus nerve, which carries the parasympathetic nervous system output, is a major constituent of a neural reflex mechanism—the inflammatory reflex—that controls innate immune responses and inflammation during pathogen invasion and tissue injury [16]. Therefore, sympathetic overactivity may be associated with a pro-inflammatory state, while increased parasympathetic activity may have anti-inflammatory properties [17, 18].

Autonomic symptoms and manifestations, including resting and postural tachycardia and orthostatic intolerance, have been frequently reported by patients with POTS and dysautonomia more broadly [5, 6, 9, 10]. Other common symptoms are chronic dizziness, including orthostatic dizziness, lightheadedness, palpitations, presyncope, syncope, orthostatic intolerance, exercise intolerance, heat intolerance, cognitive dysfunction and fatigue. Gastrointestinal, respiratory, and genitourinary symptoms are reported as well [5, 6, 9, 10].

## Clinical features and evaluation of dysautonomia

Since dysfunction of the ANS can affect multiple organs and body systems, a thorough history, review of systems, and physical exam are needed to identify whether an autonomic disorder is present and which symptoms it may be causing. Although dysautonomia is not one disorder, condition or syndrome, clinicians should suspect dysautonomia in patients with the following chronic symptoms: dizziness/lightheadedness (including orthostatic), orthostatic intolerance, exercise intolerance, “brain fog” and fatigue. In a study by Shaw et al., 99% of patients reported dizziness and 97% reported tachycardia, but other symptoms such as headache, “brain fog” (or difficulty concentrating) and fatigue were also highly prevalent [10]. Orthostatic intolerance is the hallmark of autonomic disorders, but can be difficult to elicit because some patients do not explicitly report orthostatic intolerance as a clinical feature. Similarly, many clinicians do not ask about orthostatic intolerance and do not recognize it among the patient’s presenting complaints. Therefore, the patients may need to be prompted with the following questions [5]:

- Do you have any difficulty standing or walking?
- Do you feel dizzy when you stand up?
- Do you feel that your heart is racing when you sit or stand?
- Do you need to sit down frequently when you stand or walk?
- Do your symptoms change when you are standing compared to lying down?

Physical exam in patients with dysautonomia should include a 10-min stand test where both heart rate and blood pressure are measured in the supine and standing positions after standing for 3, 5, 7, and 10 min [1, 2, 6]. This test can be done by a healthcare professional and can be used as part of the diagnostic criteria for common autonomic disorders (Table 1) [1, 2]. It is important to note that blood pressure and heart rate obtained while sitting only are insufficient for the evaluation of orthostatic intolerance. A detailed physical exam in patients with dysautonomia is indicated to rule out cardiac, neurologic, rheumatologic, immunologic, and vascular findings that may point toward a more specific disease process and warrant a referral to a specialist [6]. Abnormalities on physical exam in patients with dysautonomia may include facial flushing or paleness, dry skin and/or dry mouth, joint hypermobility, dermatographia, dilated pupils, tremulousness, fine postural tremor, and mild sensory loss in the feet, hands or face [6]. Motor examination is usually normal, but could reveal give-way weakness in patients with generalized weakness and fatigue. Give-way weakness or mild unsteadiness that can be displayed on gait exam should not be mistaken for evidence of functional neurologic disorders in patients with dysautonomia [8, 19].

A thorough cardiac and neurologic exams are recommended, including assessment of pinprick and temperature sensation to help identify small fiber neuropathy, a common comorbidity of POTS and other autonomic disorders [20]. Physical exam in patients with dysautonomia may reveal acrocyanosis—a purple-bluish discoloration of the extremities—likely caused blood pooling and skin cyanosis. Acrocyanosis may also occur in patients with Raynaud’s disease, other connective tissue disorders and erythromelalgia and may point toward autoimmune etiology [21]. Assessment for joint hypermobility with the Beighton scale is warranted in patients with dysautonomia to identify hypermobility spectrum disorders (HSD) and hypermobile Ehlers-Danlos syndrome (EDS), which are highly prevalent in patients with POTS, post-COVID dysautonomia and other forms of dysautonomia [22]. Similarly, flushing, urticaria and dermographism may be present on skin examination of patients with autonomic dysfunction and mast cell activation syndrome (MCAS) [23].

## Autonomic function testing and a 10-min stand test

Quantitative autonomic function tests include a tilt table test, Valsalva maneuver, deep breathing test, quantitative sudomotor axon reflex test (QSART), thermoregulatory sweat test and a skin biopsy for evaluation of small fiber neuropathy: these tests currently represent the best available diagnostic assessment and the most comprehensive evaluation of the autonomic nervous system. Unfortunately, the number of the autonomic laboratories in the United States and other countries is limited presenting a major barrier to clinical care. To this end, a 10-min stand test or a tilt table test, in addition to a thorough examination of the cardiovascular and neurological systems, serve as important diagnostic tests in evaluation of patients with suspected dysautonomia. The procedure for a 10-min stand test is as following [6]:

- The patient should lie down quietly for 5 min. Obtain the blood pressure and heart rate using a sphygmomanometer on the left upper arm. Leave the cuff on the arm for next measurements with the patient standing up.
- With the patient standing quietly without moving or talking, obtain blood pressure and heart rate using a sphygmomanometer and cuff positioned on the left upper arm at 3, 5, 7, and 10 min of standing.
- Record patient-reported symptoms throughout the test if/when they occur.
- Caution should be exercised for highly symptomatic patients who are unable to safely stand for 10 min due to orthostatic intolerance or other disorders with impaired mobility. We recommend aborting the test if the patient reports significant symptoms in order to prevent the patient from fainting and potentially injuring themselves.

If the 10-min stand test confirms the diagnosis of POTS, NCS, OH or orthostatic intolerance (Table 1), then no further confirmation via a tilt table test is necessary. If a 10-min stand test is inconclusive or unremarkable in a patient with suspected dysautonomia, a tilt table test, in conjunction with other autonomic function tests if available, should be considered. Clinicians may also take into consideration any available patient generated data from wearable heart rate devices or monitors (Apple watch, FitBit or similar devices) or the patient’s self-obtained 10-min stand test performed at home [6]. These data may help with diagnosis of an autonomic disorder when an in-office 10-min stand test is inconclusive. Note that a 10-min stand test may provide variable results depending on the time of the day, the patient’s symptoms at the time of the appointment, hydration status, medications and other factors. Importantly, a patient does not need to meet the diagnostic criteria for common autonomic disorders each and every time an in-office stand test is performed to keep the diagnosis of dysautonomia. Similarly, if the patient met the diagnostic criteria for common autonomic disorder once, rescinding the diagnosis is not advisable when the patient continues to experience autonomic symptoms, but does not have reproducible positive results from repeated stand tests or tilt table tests. When diagnosis is uncertain, or symptoms are progressing, other cardiovascular, neurologic, gastrointestinal, and genitourinary tests and a referral to an autonomic specialist and complete autonomic function testing needs to be considered [6, 20].

## Laboratory evaluation

In some clear cases of primary autonomic disorder or dysautonomia associated with another condition, minimal diagnostic workup may suffice. However, in many patients with multi-systemic complaints and manifestations, a more thorough laboratory assessment is recommended to include a complete blood count (CBC), a comprehensive metabolic panel (CMP) and thyroid function tests (TFT), evaluation for common vitamin and nutritional deficiencies, such as iron deficiency without anemia or mild anemia and vitamin B12 deficiency as well as morning serum cortisol to assess for adrenal insufficiency and markers of inflammation and autoimmunity, such as antinuclear antibodies (ANA) and other disease-specific antibodies, erythrocyte sedimentation rate (ESR), rheumatoid factor (RF) and C-reactive protein (CRP) (6,20). More specialized tests, such as ganglionic nicotinic acetylcholine receptor antibodies, voltage-gated potassium and calcium channel antibodies, serum and urine metabolic and mitochondrial tests, supine and standing serum catecholamines and genetic tests for hereditary connective tissue disorders, mitochondrial disorder, metabolic disorders and hereditary alpha-tryptasemia, may be warranted in those patients with clinical suspicion for specific disorders associated with dysautonomia [6, 20].

## Treatment

Therapeutic approach to common autonomic disorders consists of non-pharmacologic and pharmacologic measures. Non-pharmacologic treatment consists of increased fluid intake of at least 2.5 liters per day and salt (sodium chloride) consumption via dietary and/or supplemental sodium chloride of at least 7 grams daily [6, 24, 25]. Compression garments, such as waist-high stockings and abdominal binders, can also be effective. Avoiding dehydration, prolonged sitting or standing, and medications that can exacerbate orthostatic symptoms is advised. An individualized exercise approach using supine or sitting exercise, such as swimming, recumbent bike or a rowing machine, is encouraged since many patients, especially those with systemic comorbidities, are unable to tolerate intense exercise training regimens [26]. In patients who experience severe fatigue, exercise intolerance and post-exertional malaise, pacing and gentle movements are preferred over a more traditional physical therapy [26].

Pharmacotherapy includes first-line medications such as beta-blockers, which decrease resting and postural tachycardia by inhibiting beta-1 adrenergic receptors and reducing sympathetic overactivity; fludrocortisone, a mineralocorticoid that augments retention of water and sodium and expands plasma volume; midodrine, which is an apha-1 agonist that causes vasoconstriction and increased peripheral resistance, and other commonly used medications for the orthostatic intolerance, tachycardia and fatigue, such as pyridostigmine, ivabradine, clonidine, guanfacine, modafinil, methylphenidate and others [6, 24, 25].

Studies on pharmacotherapy in patients with dysautonomia and associated systemic disorders are lacking, but beta blockers, such as atenolol 12.5 to 25 mg daily or propranolol 5 to 10 mg three to four times a day, are a good starting point, especially in patients with tachycardia and palpitations [6]. Beta blockers in low doses are generally well-tolerated, but higher doses may result in side effects, such as fatigue, orthostatic hypotension and dizziness, and therefore, high doses that are typically used for hypertension and cardiac disease may need to be avoided. Additionally, patients with asthma or significant allergies and/or comorbid MCAS usually tolerate a low dose of a cardioselective beta blocker well. Anecdotally, low doses of cardioselective beta blockers do not appear to cause or worsen depression in patients with dysautonomia. Allergies to beta blockers are rare, but reactions to excipients and inactive ingredients may happen, especially in patients with MCAS.

Midodrine is an appropriate first-line pharmacologic option, especially in patients with low blood pressure and frequent syncope and presyncope. Three to four times dosing is indicated because of its short half-life. The patient is counselled to avoid lying down within the first hour of taking midodrine, but sitting or reclining are not contraindicated. The patient is typically instructed to check their blood pressure after initiation of midodrine to make sure it is below 130/80. Midodrine may not be the best choice in dysautonomia patients with hypertension or patients with supine hypertension or labile blood pressure though with close monitoring of blood pressure, it can be used safely in patients with syncope and orthostatic hypotension regardless of baseline blood pressure.

Fludrocortisone is often utilized for treatment of dysautonomia, but can sometimes trigger a headache or cause elevated blood pressure. As with midodrine, patients are advised to check their sitting blood pressure after initiation of fludrocortisone. Checking serum sodium and potassium concentrations if low doses of fludrocortisone at 0.1 mg to 0.2 mg daily are used is not necessary unless the patient is experiencing symptoms and signs of hypernatremia or hypokalemia.

Ivabradine is a selective inhibitor of I funny-channels at the sinoatrial node and may be safe and effective in patients with POTS and IST [27, 28]. Its beneficial use has been further highlighted in patients with Long COVID, and it is currently under investigation in the NIH-RECOVER-autonomic trials [29]. Ivabradine can serve as an excellent alternative for treatment of tachycardia in patients with dysautonomia who are unable to tolerate beta blockers due to sensitivities, intolerance, allergies and adverse effects, such as hypotension and exacerbation of asthma or depression. However, access to ivabradine might be limited by the insurance companies that require a prior authorization for its use. 

In patients with dysautonomia and complaints of muscle weakness and exercise intolerance or in those with elevated blood pressure who are unable to tolerate midodrine or fludrocortisone due to baseline or supine hypertension, pyridostigmine, which does not increase blood pressure, is a good treatment choice. Additionally, it may increase colonic motility, which can be an added benefit in patients with chronic constipation—a common complaint in patients with dysautonomia and HSD/h-EDS. Stimulants, such as methylphenidate and amphetamine salts, may be utilized in patients with significant physical and/or mental fatigue as well as cognitive complaints, especially if they do not have concurrent hypertension, tachycardia or insomnia.

Interestingly, many triggers that are known to precipitate a migraine, such as hormonal change associated with menstruation, barometric pressure or exposure to certain foods or environmental triggers, can also exacerbate autonomic and mast cell-associated symptoms in patients with the triad of dysautonomia, HSD and MCAS so avoidance of these triggers, if at all possible, is recommended. Additionally, minimizing prolonged motionless sitting and standing and avoiding or reducing over-exertion, heat, dehydration, hypoglycemia, severe physical and mental stress, sleep deprivation and recurrent infections is encouraged because patients with dysautonomia tend to be sensitive to various external and internal stimuli that can active the ANS and lead to exacerbation of symptoms.

In general, treatment approach to patients with common autonomic disorders begins with implementation of the non-pharmacologic therapies consisting of plasma volume expansion via increased water and sodium consumption, utilization of compression garments, supine and recumbent exercise and avoidance of aggravating factors. However, in many patients, non-pharmacologic therapy alone may be insufficient to improve symptoms, and therefore, a medication should be initiated. In the age of social media, artificial intelligence platforms and easily accessible medical information, many patients had already implemented a variety of non-pharmacologic therapies by the time they seek help from a healthcare professional. To this end, there should be a low threshold to initiate a medication for dysautonomia at the first encounter when a patient presents with chronic and disabling dysautonomia symptoms despite already utilizing non-pharmacotherapy. A delay in initiating pharmacotherapy often leads to prolonged and unnecessary suffering from untreated symptoms and is best avoided to optimize patient-centric care and outcomes.

In some patients with medication-resistant POTS or in those with severe nausea, vomiting or other gastrointestinal symptoms, intravenous saline can be used effectively on as-needed or intermittent basis [30]. Noninvasive vagus nerve stimulation, which is an effective treatment for migraine and cluster headache, may be considered for POTS due to its potential anti-inflammatory effect via activation of the vagus nerve and reduction of proinflammatory cytokines [31]. A gluten-free diet may be helpful, at least in a subset of patients with dysautonomia [32], and low-dose naltrexone might be beneficial for patients with dysautonomia, chronic pain and Long COVID [33–35].

Finally, immunotherapy is emerging as potentially effective in severe, treatment-refractory patients with POTS, post-COVID dysautonomia and other forms of dysautonomia and is considered standard therapy for autonomic disorders such as autoimmune autonomic ganglionopathy (AAG) and autoimmune autonomic neuropathies (AAN) in general [36–38]. One small randomized controlled trial did not show significant efficacy of IVIG over albumin in patients with POTS, but the study had significant limitations and was likely underpowered [39, 40]. IVIG is FDA-approved for treatment of acute and chronic inflammatory demyelinating polyneuropathies and myasthenia gravis, which may be associated with dysautonomia, as well as certain autoimmune disorders and common variable immunodeficiency, all of which are more prevalent in patients with POTS than the general population [14].

## Future direction

As the field of autonomic disorders evolves so do the classification system and approach to understanding and categorizing not only autonomic disorders, but also autonomic dysfunction (i.e. dysautonomia) that does not constitute a disorder based on the current diagnostic criteria. Strict criteria for autonomic disorders diagnosis and classification systems that exclude autonomic dysfunction in the context of systemic disease are not reflective of emerging science and global healthcare challenges or initiatives. With advancement in our understanding and knowledge of autonomic disorders [41], diagnostic criteria need to expand to encompass possible biomarkers and diagnostic tests that are currently under investigation. Our short-term goals should include investment of resources in education, research and availability of advanced autonomic centers and Long COVID clinics that are capable of providing diverse therapeutic options via multidisciplinary patient-centric care.

With COVID-19 pandemic, many millions of people worldwide currently experience dysautonomia as part of post-acute sequelae of SARS CoV-2 infection, which presents an enormous healthcare and economic burden [42]. Easily accessible diagnostic tests, practical and clinically relevant diagnostic criteria and effective therapies are urgently needed to diagnose and treat dysautonomia and many millions of people affected globally. Internal medicine and primary care physicians, neurologists, cardiologists, immunologists, rheumatologists and physiatrists are uniquely positioned to diagnose and treat patients with dysautonomia accurately and comprehensively. Recognition of symptoms and signs suggestive of dysautonomia is the first step to diagnosis and implementation of appropriate treatment approaches. A missed diagnosis or misdiagnosis can mean years, if not decades, of chronic and debilitating symptoms and suffering for patients, their families and society as many people with dysautonomia are young to middle-aged and in the prime of their lives. Due, in part, to COVID-19 and numerous initiatives led by professional societies and patient organizations, the diagnostic delay has been reduced from 7 years to approximately 2–3 years or less, but there is still a significant lack of awareness, education and training on how to diagnose and treat dysautonomia among healthcare practitioners and how to communicate effectively with patients living with these disabling complex conditions [43]. We can no longer consider autonomic disorders rare or esoteric: dysautonomia is a common manifestation of systemic disease and must be incorporated in the education of medical students, residents and physicians of all specialties, as part of the evaluation and management of complex patients.

## Data Availability

No datasets were generated or analysed during the current study.
